# Effects of Steel Slag on the Hydration Process of Solid Waste-Based Cementitious Materials

**DOI:** 10.3390/ma17091999

**Published:** 2024-04-25

**Authors:** Caifu Ren, Jixiang Wang, Kairui Duan, Xiang Li, Dongmin Wang

**Affiliations:** School of Chemical and Environment Engineering, China University of Mining and Technology, Beijing 100083, China; rencaifu2015@163.com (C.R.); duankairuijy@126.com (K.D.); lixiangqlmzls@163.com (X.L.)

**Keywords:** steel slag, solid waste-based binder, hydration, strength, microstructures

## Abstract

Aiming to enhance the comprehensive utilization of steel slag (SS), a solid waste-based binder consisting of SS, granulated blast furnace slag (BFS), and desulfurization gypsum (DG) was designed and prepared. This study investigated the reaction kinetics, phase assemblages, and microstructures of the prepared solid waste-based cementitious materials with various contents of SS through hydration heat, XRD, FT-IR, SEM, TG-DSC, and MIP methods. The synergistic reaction mechanism between SS and the other two wastes (BFS and DG) is revealed. The results show that increasing SS content in the solid waste-based binder raises the pH value of the freshly prepared pastes, advances the main hydration reaction, and shortens the setting time. With the optimal SS content of 20%, the best mechanical properties are achieved, with compressive strengths of 19.2 MPa at 3 d and 58.4 MPa at 28 d, respectively. However, as the SS content continues to increase beyond 20%, the hydration process of the prepared binder is delayed. The synergistic activation effects between SS and BFS with DG enable a large amount of ettringite (AFt) formation, guaranteeing early strength development. As the reaction progresses, more reaction products CSH and Aft are precipitated. They are interlacing and overlapping, jointly refining and densifying the material’s microstructure and contributing to the long-term strength gain. This study provides a reference for designing and developing solid waste-based binders and deepens the insightful understanding of the hydration mechanism of the solid waste-based binder.

## 1. Introduction

Blast furnace slag (BFS) and steel slag (SS) are two primary solid wastes in the iron and steel metallurgy industry [1]. In contrast to the successful application of BFS in the cement and concrete industry, steel slag utilization needs large progress. Take 2021 as an example: the crude iron and steel production in China was 868.57 million and 1032.79 million tons, respectively, which led to over 304 million tons of BFS and 123.93 million tons of steel slag discharge, respectively [2,3]. The utilization ratio of SS in China is only 29.5%, leading to resource waste and environmental pollution due to the large amounts of discarded SS [4,5].

Steel slag is a calcium silicatic material, with a CaO range of 38–48%, and it has a SiO_2_ range of 11–20% in chemical compositions. The Fe in steel slag is in the form of steel, FeO, and iron-bearing minerals. A lot of researchers indicate that the chemical and mineral compositions of steel slag are similar to Portland cement clinker, containing large amounts of cementitious minerals, such as C_2_S, C_3_S, and C_4_AF, which makes it potentially useful in cementitious materials [6,7]. Besides the promising application in the cement and concrete field, steel slag has also been used in road aggregates [8,9,10], carbonized minerals [11,12,13,14], fluxing agents [15,16,17], fertilizer [18], and ceramics making [19,20] due to its hard and wear resistance, higher iron content, and CO_2_ reactive characteristics, etc.

Solid waste-based cementitious materials feature non-primary resource consumption and low manufacturing energy characteristics. The synergistic activation effects between different solid wastes provide the feasibility to produce binders comparable to Portland cement binders: especially, steel slag consisting of hydraulic minerals like C_2_S, C_3_S, C_4_AF, etc. Zhao et al. studied the self-cementitious property of steel slag powder blended with gypsum. The hydration products of C-S-H gel, ettringite, and Ca(OH)_2_ were observed. The steel slag–gypsum paste exhibited compressive strength as high as 43.2 MPa at 180 days of curing. However, the steel slag–gypsum composites presented slow and lower strength gain at early ages. Therefore, the incorporation of high reactivity materials, for example, blast furnace slag, is expected to improve the solid waste-based binders’ early age strength [21,22,23,24].

Song et al. [25] reported a binder with an SS:BFS:DG ratio of 30:58:12; the obtained solid waste concrete possessed satisfactory mechanical properties, and the pH value determined from the pore solution of the binder first decreased and then increased over the curing ages. Chen et al. [26] systematically investigated the ternary systems of β-HPG (β-hemihydrate phosphogypsum)-GBFS-SS. An optimal mix proportion for β-HPG-GBFS-SS of 60-30-10 was found to give the best mechanical properties. Huang and Lin investigated the phosphogypsum–steel slag–granulated blast furnace slag binder system with and without limestone. A binder with compressive strength over 40 MPa at 28 days was observed with a mixture PG:SS:BFS:LS of 45:10:35:10. Steel slag was found to play the role of activator. However, excess steel slag would lead to a volume stability issue. Ma et al. [6] studied the effect of SS content on the strength of solid waste-based concrete, and the results showed that the contribution of SS powder to the compressive strength of concrete during the early hydration stage is less than that of BFS. The formation of AFt provides the early strength of concrete. In the later stages of hydration, AFt and C-S-H gel intertwine to form a dense structure, which is beneficial for consecutively increasing the compressive strength of concrete with time.

In summary, when slag is hydrated alone, there is no crystalline product and only a small amount of gel-like hydrate is formed. This is because the slag undergoes a hydrolysis reaction, and the Ca^2+^ and OH^−^ ions produced can combine with the silicate or aluminosilicate depolymers of the slag. It forms hydrated calcium silicate gel or hydrated calcium aluminosilicate gel. The formation of this gel allows the reactants to quickly leave the surface of the slag, and so on, so that the slag is continuously hydrated. Steel slag hydration alone will produce a certain amount of calcium hydroxide. This is because steel slag is rich in a large number of divalent metal cations, mainly Ca^2+^. After the divalent metal cations are hydrated, the alkalinity of the liquid phase increases rapidly until equilibrium state; in some positions rich in divalent metal cations, the liquid phase can reach the solubility product of calcium hydroxide, so calcium hydroxide is precipitated. However, since there is no further reaction after that, Ca^2+^ and OH^−^ can be consumed ions, and hydration products gradually wrap around the steel slag particles, making the reaction progress slowly [27].

The hydration of a steel slag–BFS composite would consume calcium hydroxide formed by steel slag hydration and form calcium silicate gel products. However, the hydration degree would be very limited. The introduction of gypsum would facilitate both steel slag and BFS hydration. However, the lower aluminum content in steel slag would result in fewer ettringite products, which mainly contribute more significantly to the early strength gain of solid waste-based binders. And, the absence of calcium hydroxide in the slag–gypsum system would also delay the early strength gain of binders. So, the combination of steel slag and BFS would provide more aluminum for ettringite formation on one hand. On another hand, the calcium hydroxide by steel slag hydration would elevate the pH of the system, favoring the strength gain of binders. However, little research has concentrated on the synergistic hydration of steel slag and BFS under gypsum activation currently. That forms the emphasis of this study.

In this study, the synergistic hydration of steel slag and BFS under gypsum activation were systematically investigated by changing the SS:BFS ratio. The reaction kinetic, compressive strength, and microstructure evolution of the prepared solid waste-based cementitious materials were studied. The aim is to provide experimental data and a theoretical basis for the resource utilization of SS, which has significant implications for the sustainable development of building materials.

## 2. Materials and Experimental Methods

### 2.1. Raw Materials

**Figure 1:**
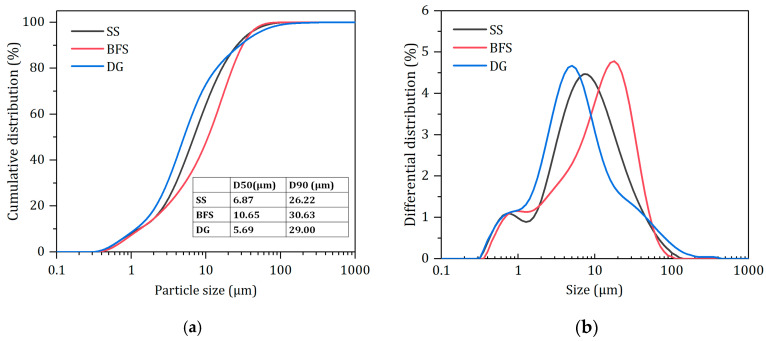
Particle size distribution of raw materials. (**a**) Cumulative distribution. (**b**) Differential distribution.

The steel slag (SS) and BFS both originate from a steel company in Guangxi province of China, with the steel slag produced by the converter hot-stewing process. The desulfurization gypsum (DG) is a by-product from a power plant in Fuyang, Anhui province of China. The specific surface areas of the SS, BFS, and DG (measured by the Blaine method) are 460 m^2^/kg, 438 m^2^/kg, and 640 m^2^/kg, respectively, with their particle size distributions shown in Figure 1. As illustrated, the SS, BFS, and DG have similar particle size distributions, with corresponding median particle sizes (D_50_) of 6.87 μm, 10.65 μm, and 5.69 μm, respectively. They also feature multi-level particle size gradations (0.1~1 μm, 1~20 μm, and 20~200 μm), which are advantageous for the synergistic hydration and filling effects of particles in different size ranges [28,29].

**Figure 2:**
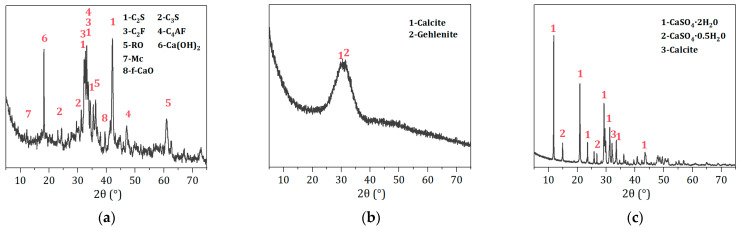
XRD patterns of raw materials. (**a**) XRD pattern of SS. (**b**) XRD pattern of BFS. (**c**) XRD pattern of DG.

**Figure 3:**
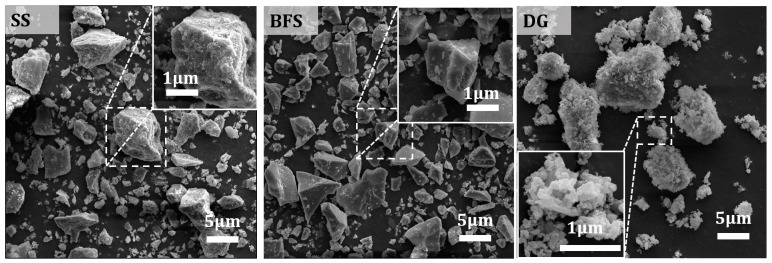
SEM morphologies of raw materials.

Table 1 and Figure 2 show the chemical and phase compositions of the raw materials. As calculated, the SS has a Mason alkalinity coefficient M = ω(CaO)/[ω(SiO_2_) + ω(P_2_O_5_)] = 2.65, which classifies it as tricalcium silicate slag. The main phases in the SS here include C_2_S, C_3_S, C_4_AF, and RO phase, and a minor quantity of Ca(OH)_2_. Rietveld quantitative analysis shows that the C_2_S phase content in the SS is 20.8%. Furthermore, the XRD pattern of the BFS shows a broad hump between 25 and 35°, indicating that BFS is primarily composed of a glassy phase with the amorphous content reaching 97.8%. In addition, it contains a tiny amount of calcite and gehlenite, with its 28-day activity index reaching 108%. The DG primarily comprises CaSO_4_·2H_2_O, which accounts for 93.2%. It also contains a minor proportion of CaSO_4_·0.5H_2_O and calcite. The presence of CaSO_4_·0.5H_2_O could be attributed to the high temperatures during the grinding process, leading to the loss of crystalline water. The microstructure of the raw materials, as shown in Figure 3, reveals that SS and BFS exhibit irregularly shaped grains with clean fracture surfaces, which is associated with their production using a vertical-roller-mill grinding process. The surface of the desulfurization gypsum is loose and porous, with small particles adhering to the surface of larger ones. Moreover, columnar or cluster-like CaSO_4_·2H_2_O crystals can be clearly observed upon magnification.

### 2.2. Sample Preparation

Based on the preliminary experimental results [30], the content of DG is fixed at 15%, while the SS is designed to be 0%, 20%, 40%, and 60% of the whole material, and the residue part belongs to BFS. The water-to-binder ratio is set at 0.35. The mix proportion design of the solid waste-based cementitious materials is shown in Table 2. 30 mm × 30 mm × 30 mm iron molds are used to cast the pastes for the phase composition and microstructure evolution examinations. The specimens are de-molded after 1 day and then cured in water at a constant temperature of (20 ± 1) °C until the target ages.

### 2.3. Experimental Procedure

#### 2.3.1. Setting Time

The setting time test is conducted following GB/T 1346-2011 [31].

#### 2.3.2. Compressive Strength

Mechanical property testing is carried out on standard mortars with a water-to-cement ratio of 0.5 and a binder-to-sand ratio of 1:3, according to GB/T 17671-2021 [32]. The tested specimens are in the form of 40 mm × 40 mm × 160 mm prisms.

#### 2.3.3. pH Value Determination

For the pH value test, 20 g powders of solid waste-based cementitious material are added to 200 mL of deionized water and stirred for 4 h, 8 h, 12 h, 24 h, 48 h, and 72 h, respectively. After each interval, 5 mL of the solution is extracted for centrifugation, and the pH of the supernatant is measured using a pH meter (RayMag, Shanghai, China).

#### 2.3.4. Isothermal Heat Responses

The heat of hydration is tested using a TAM air isothermal calorimeter (Waters^TM^, Milford, MA, USA) to monitor the reaction process of the paste over 7 days, with a water-to-binder ratio of 0.4. The sample is stirred for 30 s before placement in the sample cell, and then the heat flow and cumulative heat release are automatically recorded.

#### 2.3.5. Characterization

Samples of the hardened paste, hydrated from 3 to 90 days, are taken to examine the phase compositions and microstructures. Those samples are soaked in anhydrous ethanol five times in volume to stop the hydration reaction, and the ethanol is replaced every 24 h, for a total of three times. Before conducting subsequent characterizations, the samples are placed in a vacuum oven at 45 ± 5 °C until constant weight is achieved. Part of the samples are further ground to 80 μm for XRD, FT-IR, and TG-DSC tests. Notably, thin section samples are prepared for SEM observation, and larger particles of 10–20 mm size are used for MIP porosity testing.

The XRD test is conducted using a D8 ADVANCE (Bruker, Karlsruhe, German) diffractometer, with a scanning range of 5–70° and a scanning rate of 8°/min. A JSM-6700F (JEOL, Tokyo, Japan) scanning electron microscope is used to observe the microstructure, with the sample coated with platinum for 80 s prior to the observations. FT-IR analysis is performed using a Nicolet iS5 (Thermo Scientific™, Waltham, MA, USA) Fourier transform infrared spectrometer, with a wavenumber range of 400–4000 cm^−1^ and a resolution of 4 cm^−1^. TG-DSC analysis is conducted using an STA 449F3 (Netzsch, Selb, Germany) simultaneous thermal analyzer under a nitrogen atmosphere, with a temperature range of 30–1000 °C and a heating rate of 20 °C/min. Porosity is analyzed using an AutoPore V9600 (Micromeritics, Norcross, GA, USA) mercury intrusion porosimeter, with a pressure range from 0.698 kPa to 420.58 MPa.

## 3. Results

### 3.1. Effect of SS Content on the Evolution of pH Value of Pore Solution

**Figure 4:**
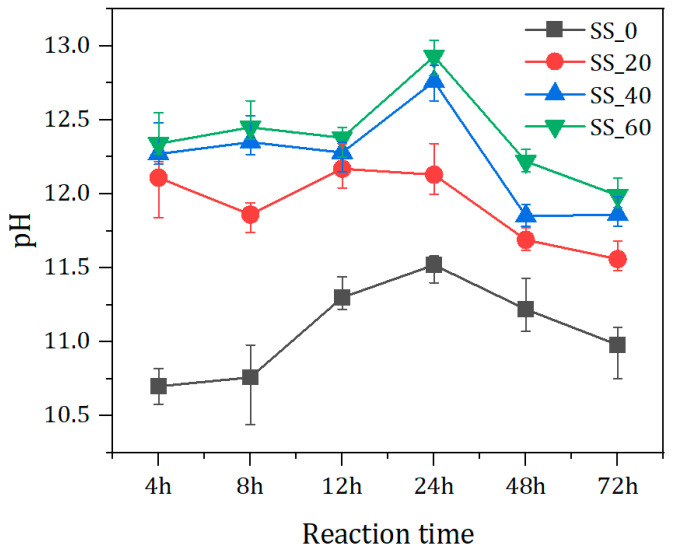
pH evolution of materials with varied CSS content over time.

Figure 4 shows the pH value changes in pore solutions of hydrated solid waste-based binders paste with different contents of SS. As seen in Figure 4, pH values of paste with SS are higher than those of paste without SS at all ages. The pH value is above 12 initially with the adding of SS. With increasing SS content, the pH value of the pore solution of the paste also rises. This is because of the quick dissolution of the small amount of Ca(OH)_2_ in SS, as shown in Figure 2a: SS consumes the calcium hydroxide. The hydration of aluminates and ferrite aluminates in the early stage also produces Ca(OH)_2_, thereby increasing the pH of the pore solution. With the progress of the hydration reaction, the pH value variations under different SS contents differ slightly. For samples without SS addition, the pH of the paste is lowest. With the dissolution of the BFS, the pH value of the paste increases, reaching a maximum value of 11.52 at 24 h. Afterward, it gradually decreases due to the formation of the hydrates consuming OH^−^ ions, as depicted in Equations (1) and (2). However, in the case of 20% SS additions, the pH of the paste first decreases, then increases, and subsequently decreases. At 12 h, the maximum is 12.17, which is determined concurrently by the dissolution and hydration of SS that produces OH^−^ and the consumption of OH^−^ by slag dissolution. Meanwhile, the consumption of OH^−^ promotes the hydration of SS, showing a temporary increase in pH value during this stage. Moreover, as the SS content is higher (40% and 60%), with the reduction in BFS content, the pH increases firstly, and then decreases. The final pH of the paste can reach up to 12.93 within the testing time scale.

(1) $$6Ca^{2+}+2Al{\left(OH\right)}_{4}^{-}+3SO_{4}^{2-}+4OH^{-}+26H_{2}O\;\to 3CaO\cdot Al_{2}O_{3}\cdot 2CaSO_{4}\cdot 32H_{2}O\;\left(AFt\right)$$

(2) $$2Ca^{2+}+AlO_{2}^{-}+OH^{-}+SiO_{2}\to \;CASH$$

### 3.2. Effect of SS Content on the Setting Times of Pastes

**Figure 5:**
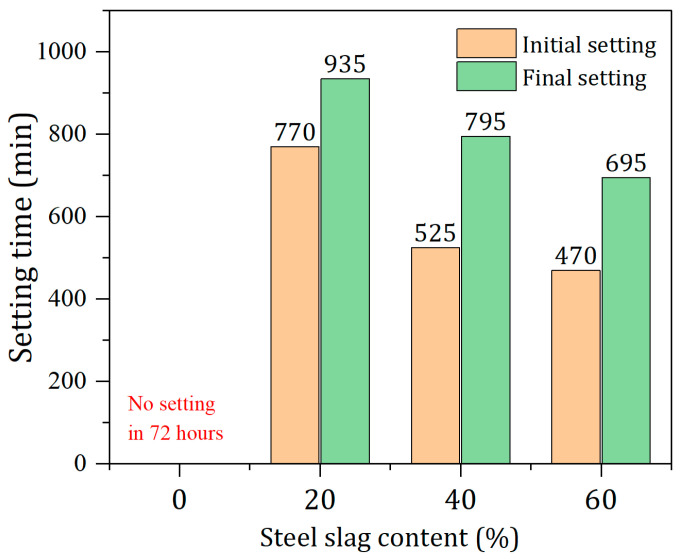
Initial and final setting times of materials with different SS contents.

The setting times of solid waste-based cementitious materials are shown in Figure 5. From Figure 5, it can be seen that with increasing SS content, both the initial and final setting times are shortened. The samples without SS are not set within 72 h and remain unhardened. According to the results in Section 3.1, the pH of the paste without SS is the lowest, which is not conducive to the early formation of hydration products and results in a longer time requirement for setting. Increasing SS content raises the pH in prepared pastes, which is favorable for accelerating the dissolution of the BFS. Then, a large amount of active silicon and aluminum substances are released from the BFS, producing many more hydration products in the early stage. This is manifested as a reduction in setting time. Specifically, when the SS content is 20% and 60%, the initial setting times are 770 min and 470 min, respectively, while the final setting times reach 935 min and 695 min, respectively. The final setting time is shortened by 240 min. Compared with traditional Portland cement (initial set ≤ 390 min, final set ≤ 600 min), the setting time of the solid waste-based cementitious materials is relatively longer.

### 3.3. Effect of SS Content on the Compressive and Flexural Strengths of Mortars

**Figure 6:**
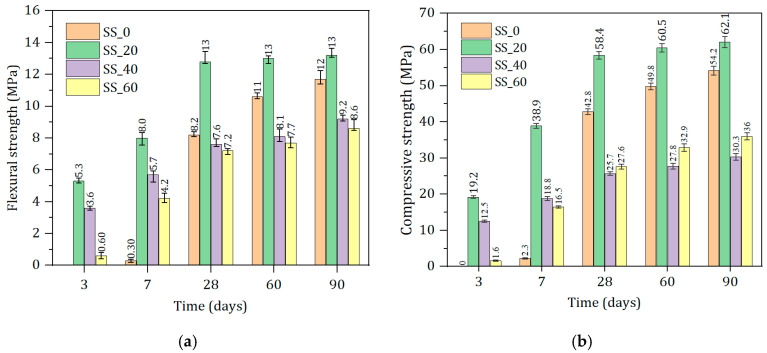
Compressive and flexural strengths of materials with different SS contents within 90 d. (**a**) Flexural strength. (**b**) Compressive strength.

Figure 6 shows that SS content significantly influences the strength of the cementitious materials, with both flexural strength and compressive strength showing similar trends, gradually increasing with the curing period’s extension. With the increase in SS content, the early strength (3 days and 7 days) first increases and then decreases. No strength gain is observed at 3 d for the group without SS, and only 2.3 MPa is gained at 7 d. The optimal compressive strength is observed with 20% SS additions, reaching 19.2 MPa (3 d) and 38.9 MPa (7 d), respectively. With 40% SS content, the strength at 3 d and 7 d are 12.5 MPa and 18.8 MPa, respectively, a reduction in value of 34.9% and 51.7% compared to the 20% SS additions group. The main reasons for the decrease in strength are the low early hydration activity of the SS and the reduction in slag content.

However, the later strength (28 days to 90 days) shows a trend of first increasing, then decreasing, and finally increasing again. With 20% steel slag content, the 28-day and 90-day strengths can reach 58.4 MPa and 62.1 MPa, respectively, with continued growth. The mechanical properties of solid waste-based cementitious materials are the worst, with 40% SS content. When the SS content is further increased (60%), the later strength slowly increases again. This indicates that as the hydration reaction proceeds, the SS can compensate for the strength loss caused by the slag reduction, ensuring the development of the later strength of the prepared cementitious material.

### 3.4. Effect of SS Content on Hydration Process and Microstructures

#### 3.4.1. Effect of SS Content on Hydration Heat

**Figure 7:**
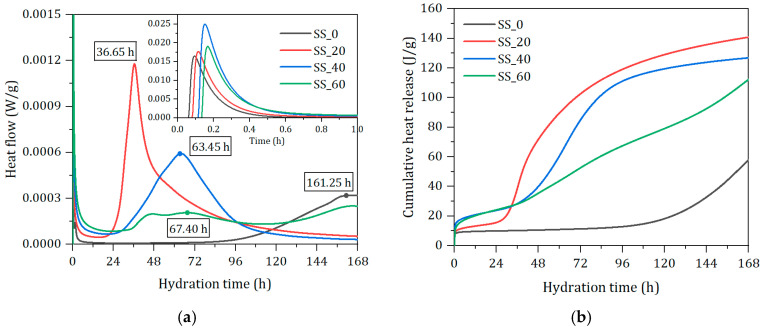
Isothermal heat responses of solid waste-based binder paste with different SS contents. (**a**) Heat flow. (**b**) Cumulative heat release.

Figure 7a,b record the instantaneous heat flow and cumulative heat release over time. The hydration process of the cementitious material can be divided into five stages [33]: rapid heat release, dormancy, acceleration, deceleration, and stabilization. Initially, the components dissolve quickly upon contact with water, forming a peak of heat release due to the release of surface energy, with the first peak occurring within approximately 0.6 h. This is followed by a lengthy dormancy period, during which the ion concentration in the pastes must reach saturation before further hydration can occur [34]. This leads to the formation of hydration products such as AFt, Ca(OH)_2_, and the nucleation of C-S-H gel, which is indicative of entering the acceleration period where a large amount of hydration products are precipitated, corresponding to the second heat release peak. With the increase in SS, the maximum heat release peaks occur at 161.25 h, 36.65 h, 63.45 h, and 67.40 h, respectively. The hydration heat peak is the most significant at 20% SS levels, but the heat release rate within 24 h is lower than that of pastes with a higher content of SS. The hydration heat release behavior of the samples without SS confirms the reasons for their longer setting time and lower strength at 7 days. In the group with a high content of SS (60%), a second hydration heat peak appears after 144 h. This is mainly due to the high content of SS, which, under desulfurization gypsum, enhances its hydration and consequently stimulates secondary hydration of the slag. The later hydration rate exceeds that of the 40% SS sample group, which is consistent with the results of the mechanical properties. The 20% SS sample group has the highest total heat release, with cumulative heat release values of 57.89 J/g, 140.80 J/g, 126.86 J/g, and 112.25 J/g. Due to the lower hydration activity of SS, its contribution to the early stage of hydration is less than that of BFS, and an increase in SS content significantly slows down the hydration process of the prepared binder.

#### 3.4.2. Effect of SS Content on Phase Composition and Chemical Characteristic

**Figure 8:**
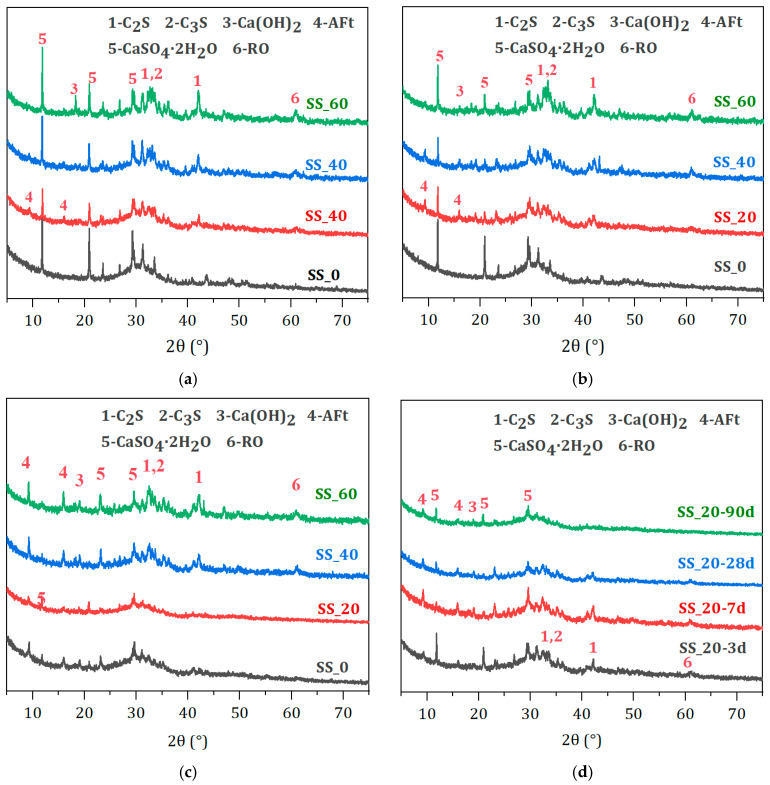
XRD patterns of hardened pastes with different SS contents. (**a**) 3 d. (**b**) 7 d. (**c**) 90 d. (**d**) 20% SS.

From Figure 8, it is found that the main hydration products in the hardened pastes are AFt and a small amount of Ca(OH)_2_, along with unreacted C_2_S, C_3_S, C_2_F, RO phase, and CaSO_4_·2H_2_O. C_2_F and RO phases are inert minerals in SS that do not participate in the hydration reaction. While C_2_S and C_3_S are active mineral components in SS, they hydrated to form C-S-H gel and Ca(OH)_2_. The C-S-H gel is amorphous. Therefore, its diffraction peaks are weak in the XRD spectra. From Figure 8a,b, it can be seen that at 3 d and 7 d, the intensity of the CaSO_4_·2H_2_O diffraction peak first decreases and then increases with the addition of SS, with the lowest peak intensity at the 20% SS incorporation group [35], indicating that more DG has been consumed in the hydration reaction, resulting in more Aft formation as a consequence. AFt diffraction peaks appear at 3 d, and with the increase in the hydration age, the AFt peaks continue to strengthen, indicating that AFt has been formed quickly in the hardened paste. For the 90-day hardened paste, the CaSO_4_·2H_2_O diffraction peak in the samples without SS is significantly weakened, while the AFt peak is enhanced considerably, indicating that a large amount of hydration products in the later stages have been formed, resulting in a substantial increase in compressive strength. As the curing age increases, the diffraction peaks of C_2_S, C_3_S, and CaSO_4_·2H_2_O weaken, indicating that the BFS has been activated under the combined action of SS and DG, deepening the hydration degree and generating more AFt and C-S-H gel. At 90 d, the AFt diffraction peaks slightly weaken, which may be related to AFt being enveloped by C-S-H in the dense structure of the paste. The samples show a low degree of crystallization of Ca(OH)_2_, which is continuously consumed to activate the BFS at a rapid consumption rate [36]. Ca(OH)_2_ hardly reaches saturation and precipitation, making its diffraction peak weak and un-distinct, becoming almost unobservable at later stages.

**Figure 9:**
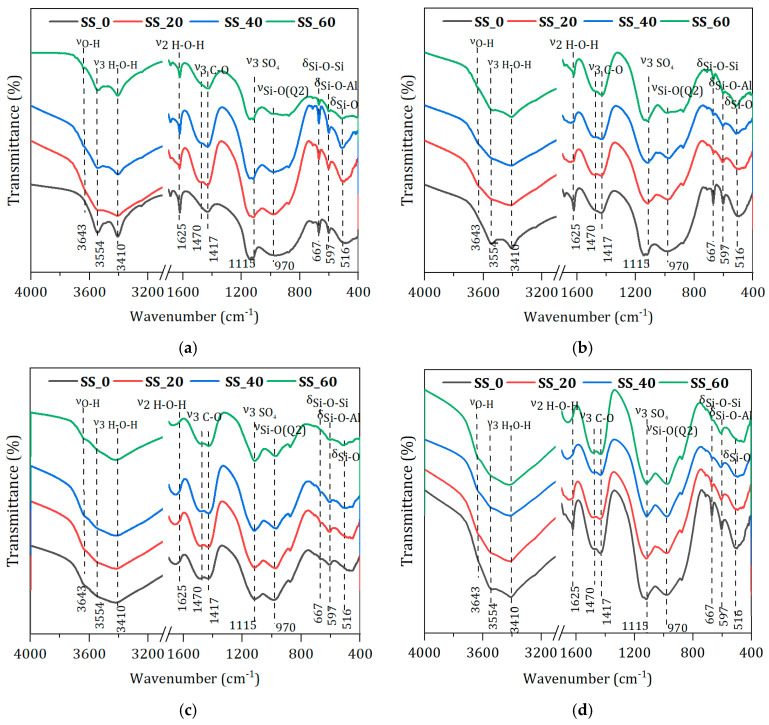
FTIR patterns of hardened pastes with different SS contents. (**a**) 3 d. (**b**) 7 d. (**c**) 90 d. (**d**) 20% SS.

Figure 9 presents the infrared spectra of hardened pastes of waste-based cementitious materials at different ages. The absorption peak at 516 cm^−1^ corresponds to the bending vibration band of the Si-O bond, while the peak at 970 cm^−1^ is attributed to the asymmetric stretching vibration of the Si-O bond in silicate tetrahedra and is characteristic of the hydrate product C-S-H gel [37]. With the variation in the amount of SS, the absorption peak at 516 cm^−1^ gradually decreases due to the ongoing disassembly of Si-O in the BFS under an alkaline environment, leading to the formation of C-S-H gel. The broadening of the characteristic peak at 970 cm^−1^ is the result of the continuous aggregation of silicate anions during the hydration process. The peak at 597 cm^−1^ represents the deformation vibration band of the Si-O-Al bond at the junction of the silicate and aluminate tetrahedra. The peak at 1115 cm^−1^ belongs to the symmetric stretching vibration peak of SO_4_ [38]. A characteristic absorption peak appears at 1115 cm^−1^ at 3 d, with the width and sharpness of the peak being affected by the amount of SS. The peak is sharper with 20% SS, indicating an early abundance of Si-O-Al bond breakage participating in the hydration reaction to form AFt. Peaks at 1470 cm^−1^ and 1417 cm^−1^ are due to the symmetric stretching vibration bands of C-O. The peaks at 1625 cm^−1^ and 3410 cm^−1^ are characteristic of the O-H vibrations of bound water in C-S-H gel and AFt. As shown in Figure 9d, the absorption peaks at both positions gradually increase with the extension of the hydration age. The peak at 3643 cm^−1^ represents the characteristic absorption peak of O-H in Ca(OH)_2_. At 20% SS content, the absorption peak is not distinctly observable at any age. This suggests that Ca(OH)^2^ is absorbed and consumed by the BFS during the hydration of the paste, resulting in the formation of a significant amount of AFt and C-S-H gel hydrates. This is consistent with the results of XRD tests.

#### 3.4.3. Effect of SS Content on the Microstructural Morphology

**Figure 10:**
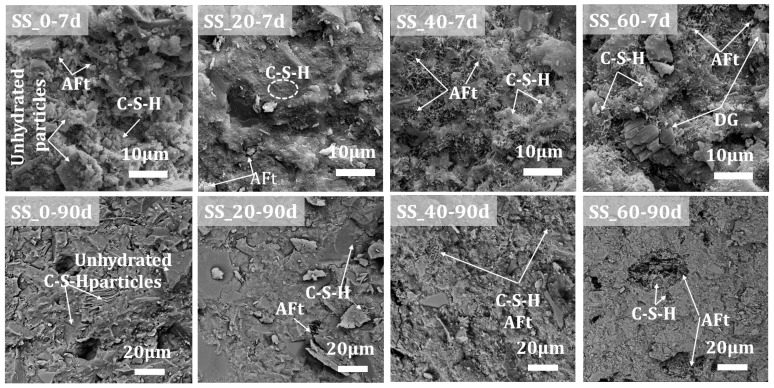
Microstructure morphologies of hardened pastes with different SS contents.

As indicated in Figure 10, the hydration products of the solid waste-based cementitious materials primarily consist of acicular or columnar AFt and amorphous fibrous C-S-H gel, with no significant presence of Ca(OH)_2_ observed, which relates to its content and crystallinity within the system. After 7 days, the hydration process in the samples without SS addition was slow, with a substantial number of unhydrated particles, fewer hydration products, and the AFt and C-S-H gel merely coating the particle surfaces without forming interconnected structures, resulting in numerous connected pores. When the SS content is 20%, a large amount of AFt and C-S-H gel hydration products interconnect the raw material particles, creating a denser structure with indistinct boundaries, which explains the superior early mechanical performance. As the SS content increases further, the hydration products overlap more, significantly increasing the interconnecting porosity and reducing the characteristics of the C-S-H gel; in the 60% SS sample group, a large amount of unhydrated DG is observed, indicating a relatively lower degree of hydration. At 90 days of hydration, for the samples without and with 20% SS addition, the internal C-S-H gel forms interconnected structures through continuous hydration, gradually resulting in a denser hardened paste structure with embedded AFt, especially for the non-SS-added sample group, which shows a significant increase in structural density compared to 7 days, consistent with the improvement in mechanical properties. With an increased SS addition (40% and 60%), due to the participation of more SS in hydration, a large amount of AFt and C-S-H grow interlaced within the pores, with AFt crystals significantly increasing in size, forming short columnar shapes and interlocking to create a complete network structure, which compensates to some extent for the early microstructural defects of being loose and porous, but still with some porosity remaining, explaining the greater increase in mechanical performance at later stages for high steel slag content solid waste-based cementitious materials.

#### 3.4.4. Effect of SS Content on Pore Characteristics

**Figure 11:**
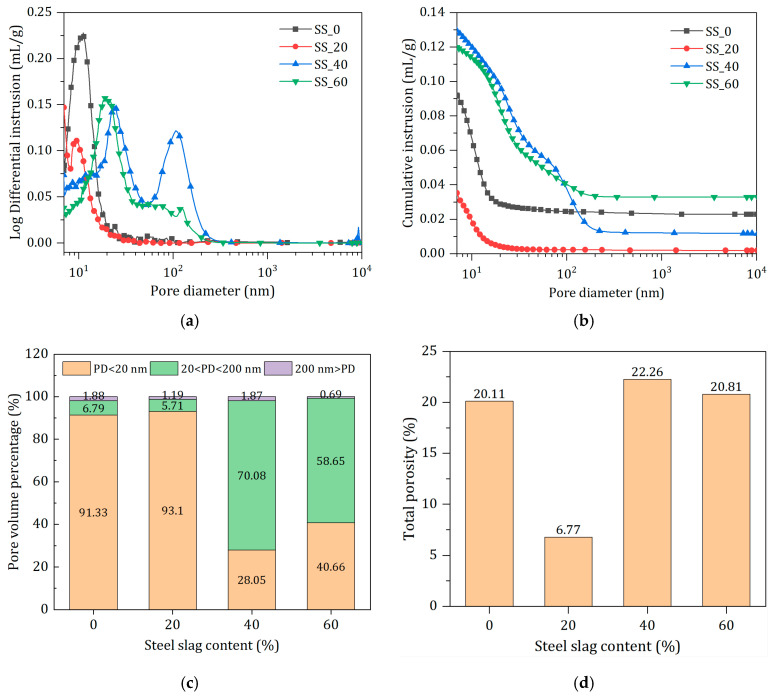
Pore characteristics of hardened pastes with different SS contents at 90 d. (**a**) Pore size distribution. (**b**) Cumulative intrusions. (**c**) Pore volume. (**d**) Porosity.

Figure 11 shows the pore size distribution and pore structure parameters of waste-based cementitious materials after 90 days of hydration. In Figure 11, the most prominent peak in the differential pore size distribution corresponds to the most probable pore size, which occupies the largest proportion of the total pore volume [39]. The order of SS content from low to high is as follows: 20% < 0% < 60% < 40%, which is consistent with the development pattern of the later strength of waste-based cementitious materials. For the 20% SS incorporations, the main pore size distribution is between 0 and 20 nm, with the most probable pore size at around 9 nm; the presence of two peaks at 40% dosage indicates a highly discontinuous internal pore distribution. The cumulative mercury intrusion volume reflects the internal pore volume (mainly open pores) [40]; as seen from Figure 11b, the pore volumes are in the order of 20% < 0% < 60% < 40%. Microscopic test results show that the hydration degree of the 20% SS hardened paste is higher, producing more hydration products, filling the internal pores [41] and resulting in a denser microstructure, thus presenting the smallest, most probable pore size and total mercury intrusion volume. Pores within the hardened paste can be categorized into three types: pores smaller than 20 nm, 20~200 nm, and larger than 200 nm, with their respective pore contents shown in Figure 11c, and the porosity of waste-based cementitious materials listed in Figure 11d. It can be observed from the figures that the proportion of harmless pores (<20 nm) in the 20% SS hardened paste reaches 93.1%; compared with the 40% and 60% steel slag sample groups, there is an increase in small pores and a decrease in large pores, which is consistent with the porosity observed via SEM. The porosity of the 20% SS hardened paste is only 6.77%, a reduction of 66.3% compared to the sample without steel slag (20.11%). This is a direct cause of the best mechanical performance and reaffirms the vital role of a reasonable dosage of steel slag in the hydration process of waste-based cementitious materials.

#### 3.4.5. Effect of SS Content on Hydration Degree

**Figure 12:**
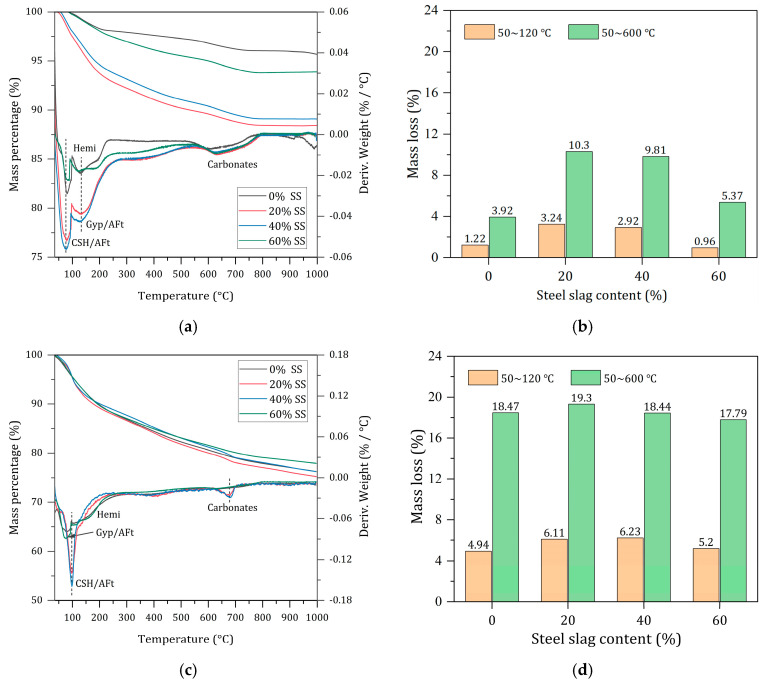

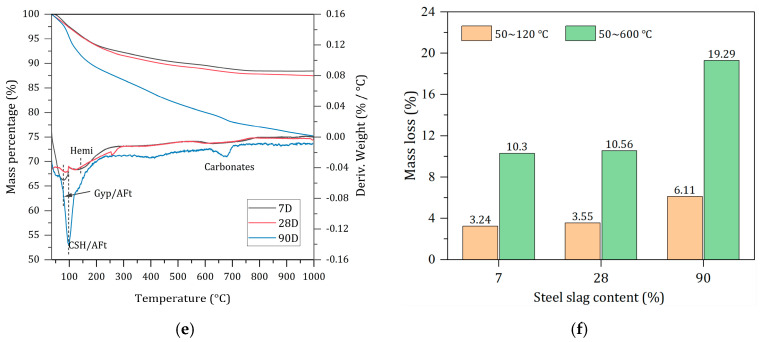
TG-DTG results of hardened pastes with different SS contents at 90 d. (**a**) TG-DSC curves of 7 d hydrated samples. (**b**) Mass loss of 7 d hydrated samples. (**c**) TG-DSC curves of 90 d hydrated samples. (**d**) Mass loss of 90 d hydrated samples. (**e**) TG-DSC curves of the group with 20% SS additions. (**f**) Mass loss of the group with 20% SS additions.

AFt, C-S-H gel, and a small amount of unreacted gypsum have been detected through tests such as XRD and FT-IR. AFt dehydrates at around 90~120 °C [42], while gypsum dehydrates at around 140 °C [43]; C-S-H has a wide dehydration temperature range (around 50~600 °C) due to the presence of various types of water within its structure. However, the main weight loss peak for C-S-H is around 120 °C, which can overlap with AFt and gypsum, especially AFt. Therefore, this paper divides the weight loss temperature ranges into 50~120 °C and 50~600 °C to quantitatively calculate bound water content in hydration products, thus better evaluating the degree of hydration of solid waste-based cementitious materials [44,45]. Figure 12 shows the TG-DTG curves and weight loss content of solid waste-based cementitious material hardened paste at different ages and mix ratios. From Figure 12a, it can be seen that for the 7-day hydrated hardened paste, the DTG peaks for AFt and C-S-H gel dehydration are near 112 °C, and the peak for gypsum dehydration is at 138 °C. When SS is not added, there are no significant peaks for AFt and C-S-H gel, indicating a lower quantity of hydration products and a lower degree of reaction. The samples with 20% and 40% SS did not show significant gypsum dehydration peaks, indicating that the gypsum content in the system is low, and its participation in the reaction has enhanced the hydration process. According to Figure 12b, the weight loss content within the temperature ranges of 50~120 °C and 50~600 °C initially increases and then decreases with the increase in steel slag content, with the maximum weight loss occurring at 20% steel slag, reaching 3.24% and 10.30%, respectively, indicating the formation of more hydration products. Figure 12c shows that as the hydration process continues, gypsum is continuously consumed. For the 90-day hydrated hardened paste, no significant gypsum dehydration peaks are present in the DTG curves of all samples. In Figure 12d, the total weight loss content at 50~600 °C initially increases and then decreases with the change in SS content, with the highest at 20% SS, reaching up to 19.3%, indicating the highest degree of hydration. It was also found that there were almost no peaks for Ca(OH)_2_ around 450 °C in the DTG curves of 7 days and 90 days, consistent with XRD and SEM analysis results. Figure 12e,f show the TG-DTG curves and weight loss changes of the 20% steel slag samples at different hydration ages, indicating that the weight loss content increases with the extension of hydration age, with a significant increase in bound water content from 28 days to 90 days, and the total weight loss increasing from 10.56% to 19.29%, an increase of 82.67%. This suggests that the solid waste-based cementitious materials continue to generate a large amount of AFt and C-S-H gel hydration products in the later stages to ensure the continuous development of their mechanical properties. Additionally, the endothermic peak near 700 °C is caused by the decomposition of a small amount of calcium carbonate, and the noticeable exothermic peak around 880 °C corresponds to the phase transformation of the C-S-H gel, converting into β-wollastonite [46,47].

In the SS-BFS-DG solid waste-based cementitious material system, in the early stages, compounds such as C_2_S, C_3_S, and C_4_AF in the SS undergo hydration reactions to form C-S-H and Ca(OH)_2_, thereby raising the pH value of the paste and providing an alkaline environment for the system. Under the joint action of DG, the tetrahedra of silicon and aluminum in the slag dissociate, releasing more reactive silica and alumina, which lead to the formation of AFt and C-S-H gel. Furthermore, the hydration reaction of the BFS reciprocally enhances the continuous hydration of the SS, while consuming the DG, and the synergistic hydration of the three components leads to the generation of a significant amount of hydration products in the later stages of the cementitious materials. AFt and C-S-H gels interlace and overlap, filling the pores and forming a dense microstructure, thereby enhancing the performance of the solid waste-based cementitious materials. It is worth noting that due to the low early reactivity of the SS, a large substitution amount significantly delays the hydration process of the cementitious material system, reducing the degree of hydration. Therefore, it is essential to control the amount of SS added to prepare solid waste-based cementitious materials with excellent performance. Additionally, according to the findings of this study, solid waste-based cementitious materials have shortcomings, such as long setting times and low early strength; therefore, the introduction of accelerators as admixtures to improve their early performance will be explored in subsequent experiments.

## 4. Discussion

The effects of SS on the hydration of BFS-DG composites have been systematically studied in this study. Steel slag, as a potential activator, has been proven sufficient in improving slag hydration degree, and performance development of solid waste-based binders. However, there is an optimal content for SS incorporation in solid waste-based binders due to the adjusting effects on the hydration of the BFS-DG composite.

For a neat BGS-DG system, the hydration of slag at an early age is very limited due to the lower alkalinity of the system. Within the early 72 h, the pH of the pore solution in the paste is not over 11.5, which is lower than the alkalinity for ettringite. As reported by [48], ettringite only precipitated in a BFS slurry with a pH over 12. Therefore, the saturation first reached is C-S-H; the formation of C-S-H consumed OH^−^ and lowered the alkalinity. The supersaturation state in the BFS-DG system is hard to reach. Thus, in the BFS-DG system, only ettringite formation is observed in the early 7 days. The binders present poor and slow strength development in the early stage.

The addition of SS elevated the alkalinity of the pore solution in two aspects: Firstly, by the dissolution of calcium hydroxide contained in SS. Secondly, the hydration of reactive C_2_S, and C_3_S in SS further provided calcium hydroxide. With 20 wt% of substitution of BFS by SS, the alkalinity of the pore solution elevated to over 12. The main hydration reaction was largely advanced. In particular, the ettringite formation was advanced and strengthened. The calorimetry responses showed that not only the hydration rate but also the overall hydration degree were largely improved. However, it was found that there is an optimal content for SS. Wang et al. [49] studied the effects of activators on the hydration process of solid waste-based binders; it was found that excess activators suppressed the hydration of BFS and the formation of ettringite, although the C-A-S-H formation was strengthened. The ettringite formation rate is much higher than C-A-S-H. So, once the saturation state has been reached, the ettringite formation is very intense and concentrated. The hydration products can accumulate in a very short time and fill the space of hydrates resulting in the strength gain of the hydrate matrix. So, it is very vital to adjust the formation of ettringite in a solid waste-based binder.

Actually, the optimal activator content in solid waste is dependent on the materials used. Several activators have been studied in solid waste-based binders, like lime, clinker, and carbide residue. However, excess activator suppressed the slag dissolution and delayed the hydration process. Wang et al. [50] found that a higher content of activator favored the out-products formation. The formed C-A-S-H layer suppressed the diffusion of the ions further. The overall hydration degree was limited with excess activators. In the case of this study, minerals like C_2_S and C_3_S in SS are more reactive than BFS. So, if too much SS is added, the hydration of C_2_S and C_3_S in SS also suppressed the hydration of BFS, resulting in negative effects on the performance development of the solid waste-based binder. 

## 5. Conclusions

(1) Within the range of 0% to 60% steel slag content, as the proportion of steel slag increased, the pH value of the solid waste-based cementitious material system was elevated, and the setting time was reduced. In the test group without steel slag addition, there was no setting within 72 h, indicating that steel slag played a significant role in the early hydration stage.
(2) With the optimal steel slag content of 20%, the solid waste-based cementitious materials achieved the best mechanical properties, with the compressive strengths at 3 d and 28 d reaching 19.2 MPa and 58.4 MPa, respectively. The addition of SS improved the early strength gain of solid waste-based binders compared to the reference sample without SS. However, with SS beyond 20%, the compressive strength declined in large scale due to decrease in BFS.
(3) The hydrated solid waste-based cementitious material composed of steel slag, granulated slag, and desulfurization gypsum are primarily AFt and C-S-H gel. In the early stages, AFt primarily contributes to the strength, while in later stages, a substantial amount of AFt and C-S-H gel hydration products are formed. The interlocking and overlapping of AFt with C-S-H gel fills the pores and creates a dense microstructure, thereby ensuring the continuous increase in the strength of the cementitious material system.
(4) The addition of steel slag advanced the main hydration and contributed to more hydration products formation. The bond water contents at seven days were significantly increased with SS addition and reached the maximum with optimal content of SS. The pore structure was also refined by the addition of SS with optimal content of SS. Excess of SS resulted in the pore structure coarsening and poor volume stability of the solid waste-based binder.

## Figures and Tables

**Table 1:** The chemical compositions of raw materials (wt.%).

Materials	CaO	SiO_2_	Al_2_O_3_	Fe_2_O_3_	MgO	SO_3_	P_2_O_5_	Cl	Others
SS	40.29	13.86	6.49	22.43	9.47	0.93	1.35	0.10	5.08
BFS	45.69	23.39	15.43	0.59	9.97	1.54	0.02	0.10	5.38
DG	42.97	2.30	2.24	1.39	2.05	47.44	0.04	0.21	1.40

**Table 2:** Mixing proportions of solid waste-based material.

No.	Materials/wt.%		
	SS	BFS	DG
SS_0	0	85	15
SS_20	20	65	15
SS_40	40	45	15
SS_60	60	25	15

## Data Availability

Data are contained within the article.
